# Hiding in Plain Sight: Nebivolol Exhibits Compelling Tocolytic Properties

**DOI:** 10.1111/jcmm.13883

**Published:** 2018-09-06

**Authors:** Scott D. Barnett, Iain L. O. Buxton

**Affiliations:** ^1^ Pharmacology University of Nevada Reno School of Medicine Reno Nevada

**Keywords:** GSNO, GSNOR GSNO reductase, ADH5 nebivolol, nitric oxide, pregnancy, preterm labour, smooth muscle, S‐Nitrosoglutathione

## Abstract

Preterm birth before 37 weeks of completed gestation results in numerous health consequences for the foetus. Preterm labour leads to preterm birth in over 50% of cases, and no FDA‐approved treatment can prevent labour or help a foetus remain in the womb until term. Examination of nitric oxide mediated relaxation signaling in the uterine smooth muscle reveals a role for protein S‐nitrosation. The recent discovery of upregulated S‐nitrosoglutathione reductase (GSNOR) in spontaneously preterm labouring women has emphasized the need to explore the function of S‐nitrosation regulation in the maintenance of uterine quiescence. Here we have examined the ability of nebivolol to relax uterine smooth muscle and tested recent claims that nebivolol is a GSNOR inhibitor. In uterine smooth muscle strips from both mouse and human, nebivolol relaxes oxytocin‐induced contractions in a dose dependent manner. Our data indicates that nebivolol has no effect on GSNOR activity, nor does nebivolol inhibit thioredoxin reductase, two of the major protein denitrosylases. The ability of nebivolol to relax uterine smooth muscle is likely the combined effects of increased nitric oxide synthase activity and β3‐adregnegic stimulation.

## 1 INTRODUCTION

Spontaneous Preterm Labour (sPTL) leads to Preterm Birth (PTB). PTB remains the primary cause of neonatal morbidity and hospitalization during pregnancy,^1^ and in the United States alone one in eight infants are born prematurely, resulting in 20,000 deaths annually.^2^ Currently available drugs are not effective at delaying birth beyond 48‐hours.^3^ This observation is not surprising when we consider that tocolytics, in general, are borrowed pharmacology. That is to say, nearly every drug used to treat PTL was initially intended to treat maladies in other smooth muscle types, such as vascular, colonic and airway. Even atosiban (Tractocile^®^; Ferring Pharmaceuticals, Parsippany, NJ), a selective oxytocin‐vasopressin receptor antagonist designed specifically to mitigate uterine contractions, is not approved for use in the United States and does not reduce the risk of preterm birth beyond 48‐hours or improve neonatal outcome.^4^

The myometrium is a unique subclass of smooth muscle that exhibits a signalling exception to the dogma that global cGMP accumulation drives nitric oxide (•NO)‐mediated relaxation. It is well‐established that term human myometrium relaxes to •NO in an cGMP‐independent fashion.^5^, ^6^, ^7^ •NO is a critical mediator of uterine quiescence; however, •NO's most pervasive role in the myometrium is through protein S‐nitrosation (SNO),^8^ rather than cGMP accumulation. S‐nitrosoglutathione reductase (GSNOR) is an important modulator of •NO and S‐nitrosation.^9^ GSNOR is upregulated in human sPTL myometrium, and inhibition of GSNOR by N6022 can partially restore native myometrial function.^6^

At first glance, nebivolol seems like an unlikely tocolytic. Nebivolol is a third generation, FDA‐approved β1‐adregneric receptor (β1AR) antagonist commonly used for the treatment of heart failure. Nebivolol also serves a lesser known secondary function as a β3AR agonist,^10^ which facilitates vasodilation by increasing endothelial •NO‐synthase (eNOS) activity and expression.^11^ Nebivolol increases SNOs and has recently been reported as a GSNOR inhibitor,^12^ which if true would further increase •NO concentration in the cell. The •NO‐promoting effects of nebivolol makes it a candidate therapeutic for inotropic muscle conditions other than vascular smooth muscle disease, such as sPTL, where SNOs are decreased. Here, we test the tocolytic properties of nebivolol and determine if nebivolol does in fact act as a GSNOR inhibitor.

## 2 METHODS

### 2.1 Contractile studies

Strips of either human or mouse (C57BL/6J) myometrium (~0.5 × 15 mmol L^−1^) were clip‐mounted by silk thread, attached to a force transducer and isometrically stretched in an organ bath (WPI, Sarasota, FL) containing Krebs buffer. Tissues were maintained at 37°C, gently bubbled with balanced oxygen (95% O_2_, 5% CO_2_) and challenged with KCl (60 mmol L^−1^ replacing NaCl) for 3 min, followed by washout. Tissues were equilibrated for 1 h, then further challenged with oxytocin (8 nmol L^−1^), followed by washout. Samples utilizing the β3AR antagonist, SR59230A (10 μmol L^−1^), were pre‐incubated 15‐min prior to baseline recordings. Data were analyzed with LabScribe (version 3.015800, Mac OS 10.11; iWorx systems inc., Dover, NH).

### 2.2 GSNOR activity assay

GSNOR enzyme activity was determined as previously described^13^ using total protein lysate from human uterine smooth muscle tissue taken from the superior portion of the transverse incision. The lysate was prepared to a final protein concentration of 1 mg/mL in oxygen‐purged buffer containing: Tris‐HCl pH 8.0 (20 mmol L^−1^), EDTA (0.5 mmol L^−1^), NP‐40 (0.1%) and 1 mmol L^−1^ phenylmethylsulphonyl fluoride (PMSF) and equilibrated at r.t. for 10 min in the presence of NADH (300 μmol L^−1^) prior to addition of GSNO (200 μmol L^−1^). Absorbance at 340 nm (A340) was recorded at *t* = 0, 5, 10 min to ensure stability of the NADH pool prior to the addition of GSNO and/or inhibitors. N6022 (8 nmol L^−1^) (S77589: Selleck Chemicals, Houston, TX), a GSNOR inhibitor, was used to verify negligible NADH conversion to NAD+ in the presence of GSNO.

### 2.3 SC‐TR activity assay

The selenocystamine‐thioredoxin reductase (SC‐TR) assay was performed as previously described^14^ with the exception that selenocystamine was used in lieu of selenocystine as the subtrate. HEK293 cells were grown to 90% confluence and lysed with ice‐cold TE‐buffer (pH 7.5). 100 μL reaction volumes were used in costar 3396 96‐well polystyrene plates and read on a Hidex Chameleon (model 425‐106, MikroWin software ver. 4.43). Reaction mixtures consisted of: 500 μmol L^−1^ NADPH (sigma N1630), 800 μmol L^−1^ selenocystamine (sigma S0520) and 25 μg total HEK293 protein lysate, with or without 1 or 10 μmol L^−1^ auranofin (sigma A6733), in a final volume of 100 μL. Protein lysates and inhibitors were equilibrated at r.t. for 5 min prior to the start of the assay. Samples were read at 30 sec intervals for 20 min at 365 nm. Data are displayed relative to baseline activity (0‐1) over the 20‐min time‐course.

### 2.4 Analysis

All data analysis were conducted using Prism (version 7.0c for Mac OS 10.11, GraphPad Software, La Jolla, CA). Significance is defined as *P* < 0.05 using an unpaired, two‐tailed, student's *t* test, unless otherwise stated.

## 3 RESULTS

### 3.1 Nebivolol decreases uterine force and AUC

**Figure 1:**
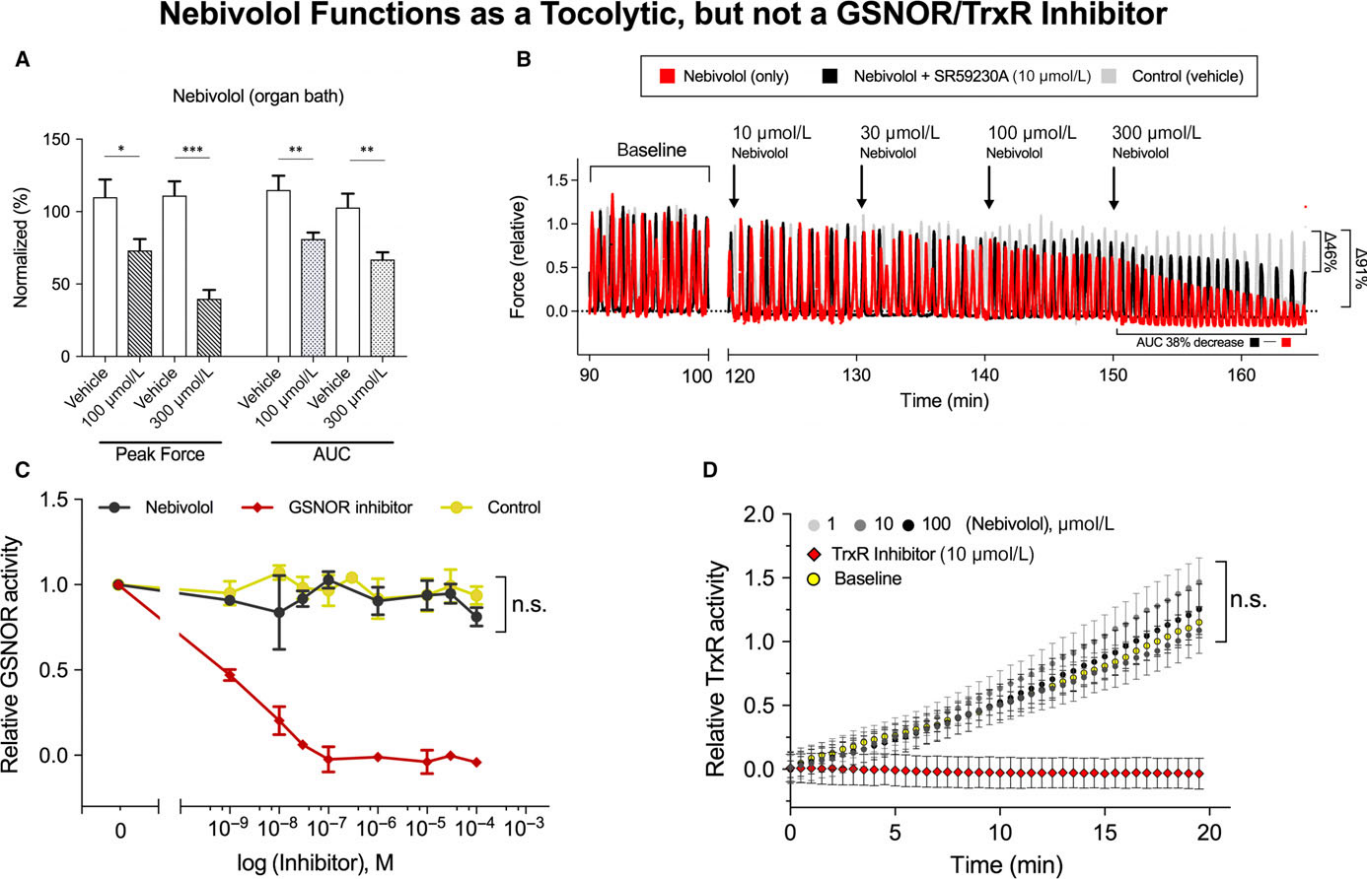
A, Oxytocin‐primed human myometrium contractile dynamics in an ex vivo myobath after a 30‐min incubation with either 100 μmol L^−1^ or 300 μmol L^−1^ nebivolol (n = 4). Peak force (100 μmol L^−1^ *P* = 0.0185, 300 μmol L^−1^ *P* = 0.0006) and area under the curve (AUC) (100 μmol L^−1^ *P* = 0.008, 300 μmol L^−1^ *P* = 0.0033) were significantly decreased at both concentrations when compared to a DMSO control. B, Oxytocin‐primed C57BL/6J mouse uterine strips were hung in a myobath and SR59230A (10 μmol L^−1^), a β3AR antagonist was added to one bath (black), 15‐min prior to baseline recording, then an accumulative dose of nebivolol (10‐300 μmol L^−1^) was administered in 10‐min increments (black/red) and compared to a DSMO control (grey). C, Relative GSNOR activity was measured after 10‐min in the presence of either nebivolol, N6022 (GSNOR inhibitor), or DMSO control (baseline), at doses between 1 and 100 μmol L^−1^. Nebivolol did not inhibit GSNOR (*P *= 0.1129). D, TrxR activity was no different from baseline after 20 min in the presence 1 μmol L^−1^, 10 μmol L^−1^ or 100 μmol L^−1^ nebivolol (n.s., n = 3), whereas 10 μmol L^−1^ auranofin (TrxR inhibitor) was significantly decreased from baseline (*P* < 0.0001, n = 3). All data presented as mean ± SEM. * *P* ≤ 0.05, ** *P* ≤ 0.01, *** *P* ≤ 0.001

In order to determine whether nebivolol acts as a negative inotropic agent, we utilized a tissue organ bath with either human or mouse myometrium. Nebivolol was applied at 100 μmol L^−1^ and 300 μmol L^−1^ to OT‐primed (8nM) term non‐labouring human (Figure 1A, TNL: N = 4), or mouse (Figure 1B), myometrium. Nebivolol decreased TNL human myometrial contractile dynamics, where both peak force (100 μmol L^−1^ *P* = 0.0185, 300 μmol L^−1^ *P* = 0.0006) and AUC (100 μmol L^−1^ *P* = 0.0080, 300 μmol L^−1^ *P* = 0.0033) were significantly decreased at either nebivolol concentration. Similarly, peak force and AUC in mouse myometrium decreased with an accumulative dose of nebivolol (1‐300 μmol L^−1^, 10‐min steps). Under conditions in which mouse tissue was preconditioned with the β3AR antagonist SR59230A (10 μmol L^−1^), the effect of nebivolol decreased by ~50% over baseline, indicating actions of nebivolol beyond of β3AR stimulation (Figure 1B).

### 3.2 Nebivolol does not inhibit S‐nitrosoglutathione reductase

Recently, researchers have suggested that because nebivolol shares structural similarities to N6022, a known GSNOR inhibitor, and because nebivolol increases endogenous •NO and total protein S‐nitrosations, nebivolol may also act as a GSNOR inhibitor.^12^ To test this, we utilized a GSNOR activity assay. Data are reported as GSNOR activity within a range between 0 and 1. NADH consumption after 10 min [GSNO (300 μmol L^−1^) + NADH (200 μmol L^−1^)] + protein lysate (1 mg/mL)) was set to a nominal value of “1”, while NADH consumption of the reaction mixture (same as above (‐)GSNO) after 10 min was set to a nominal value of “0”. Drug concentrations between 1 nmol L^−1^ and 100 μmol L^−1^ were tested and NADH consumption at 10 min was recorded as a metric of relative GSNOR activity. One‐way ANOVA analysis for the action of each candidate drug on GSNOR activity is as follows: N6022 (known GSNOR inhibitor) “F (8, 18) = 174.1” *P* < 0.0001, Nebivolol “F (7, 16) = 2.038” *P* = 0.1129 (n.s.) and baseline “F (9, 20) = 1.229” *P* = 0.3323 (n.s.). Our results indicate that nebivolol does not inhibit GSNOR (Figure 1C).

### 3.3 Nebivolol does not inhibit thioredoxin reductase

Another major contributor to •NO metabolism in the myometrium is thioredoxin reductase (TrxR). TrxR depletes •NO from the cell by reducing S‐nitrosated thioredoxin. Because S‐nitrosations are labile and the NO‐moiety can be non‐enzymatically transferred between complimentary cysteine thiols, the reduction of S‐nitrosated proteins proportionally decreases available •NO in the system. TrxR activity is measured similar to GSNOR using a selenocystamine‐thioredoxin reductase assay, except NADPH is substituted for NADH, and the substrate for this assay is selenocystamine, for which TrxR is the only known endogenous metabolizer. We determined that nebivolol (1‐100 μmol L^−1^) does not inhibit TrxR activity (Figure 1D) (n = 3 for all conditions), as analyzed with a one‐way ANOVA: “F (2, 117) = 2.694” *P* = 0.0718. As predicted, auranofin, a known inhibitor of TrxR, inhibited the enzyme in a dose dependent fashion (*P* < 0.0001; only the 10 μmol L^−1^ dose shown for clarity). These data further indicate that the tocolytic effects of nebivolol are not the result of the inhibition of •NO metabolizing enzymes.

## 4 DISCUSSION

**Figure 2:**
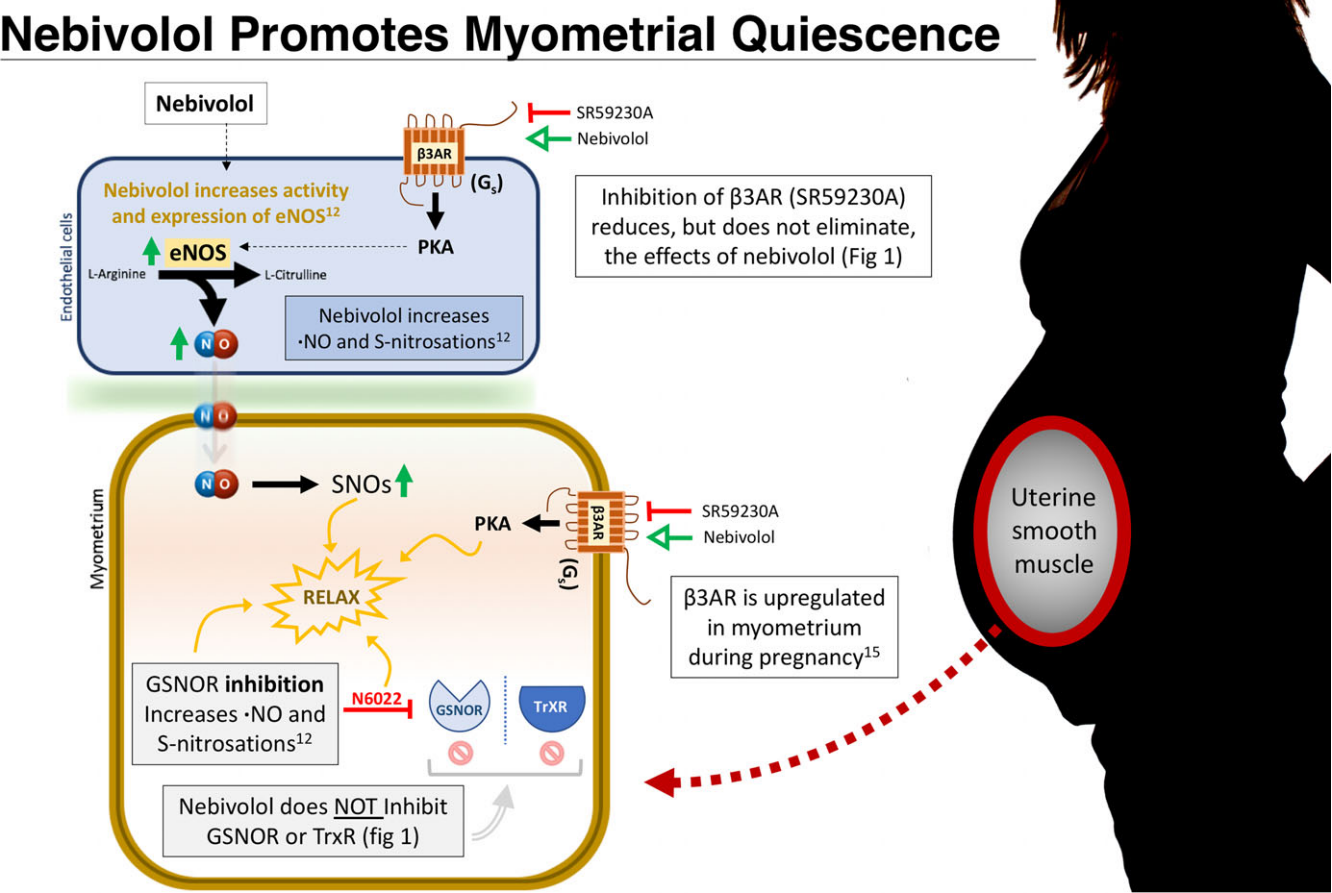
Nebivolol drives smooth muscle relaxation through multiple pathways. First, nebivolol activates β3ARs on both endothelial and myometrial cells. β3ARs are upregulated in the myometrium during pregnancy,^16^ potentially heightening this effect. Nebivolol further enhances endothelial nitric oxide synthase (eNOS) expression and activity.^11^ This resulting surge of nitric oxide increases total protein S‐nitrosations (SNOs),^12^ promoting SNO‐mediated myometrial smooth muscle relaxation.^9^ Inhibition of S‐nitrosoglutathione reductase (GSNOR) also increases nitric oxide availability, which relaxes myometrial tissue^6^; however, nebivolol does not inhibit the SNO‐metabolizing enzymes GSNOR or thioredoxin reductase (TrxR)

Our findings indicate that Nebivolol's potential as a tocolytic is most likely the result of enhanced eNOS activity, driven in part by β3AR stimulation (Figure 2). These data compliment our earlier findings that GSNOR is upregulated in some women who undergo sPTL,^6^ decreasing the endogenous intracellular •NO pool, and by extension, total protein S‐nitrosations. While nebivolol does not inhibit •NO metabolism as others have suggested^12^ (Figure 1B,C), the same functional end state is achieved through nebivolol‐mediated •NO generation. We found that nebivolol was able to decrease peak force and AUC in myometrial samples (Figure 1A) more effectively than N6022,^6^ a known GSNOR inhibitor, perhaps in part due to the limited bioavailability of N6022. The use of nebivolol as a tocolytic is further supported by the finding that β3AR is the predominant subtype in human myometrium, is upregulated during pregnancy,^15^ and direct stimulation of β3AR has been shown to promote myometrial relaxation.^16^

A conspicuous advantage to using nebivolol as a tocolytic agent is that it is already FDA‐approved. While other “borrowed” tocolytics have failed to delay PTB, nebivolol is unique in that it promotes relaxation by increasing the •NO pool, which leverages the problem of GSNOR upregulation in sPTL myometrium.^6^ That being said, β ‐blockers use during pregnancy would need to be weighed again potential adverse effects on the foetus, such as small‐for‐gestational‐age infants.^17^ While long term use would raise additional issues,^18^ these are based upon near continuous administration throughout gestation, while tocolytic use can be invisioned as a shorter treatment course. Further studies are clearly needed to establish safety and efficacy of nibivolol as a tocolytic in pregnant women.

## CONFLICT OF INTEREST

The authors confirm that there are no conflicts of interest.
